# Effect of Ajwa Dates Consumption to Inhibit the Progression of Preeclampsia Threats on Mean Arterial Pressure and Roll-Over Test

**DOI:** 10.1155/2019/2917895

**Published:** 2019-12-10

**Authors:** Ida Royani, Suryani As'ad, Nasrudin A Mappaware, Mochammad Hatta, Rabia

**Affiliations:** ^1^Department of Nutritional Science, Faculty of Medicine, Universitas Muslim Indonesia, Makassar 90231, Indonesia; ^2^Department of Nutritional Science, Faculty of Medicine, Hasanuddin University, Makassar 90245, Indonesia; ^3^Department of Obstetrics and Gynaecology, Faculty of Medicine, Universitas Muslim Indonesia, Makassar 90231, Indonesia; ^4^Department of Microbiology, Faculty of Medicine, Hasanuddin University, Makassar 90245, Indonesia; ^5^Department of Physiotherapy, Faculty of Nursing, Hasanuddin University, Makassar 90245, Indonesia

## Abstract

**Background:**

Preeclampsia is the major problem and the main leading cause of fetal and maternal mortality worldwide. The early prediction of preeclampsia in pregnant women is required to prevent the occurrence of preeclampsia. The mean arterial pressure (MAP) and roll-over test (ROT) are the combination of measurement which can be used to predict preeclampsia. On the contrary, Ajwa dates were reported to have an enormous activity which contributes to its role in improving health conditions. In this study, we aimed to evaluate the effect of daily consumption of seven Ajwa dates on prevention of preeclampsia, through MAP and ROT changes.

**Methods:**

Forty pregnant women (*n* = 40) were randomly assigned into the control group (*n* = 10) and the intervention group which received a daily intake of Ajwa dates (*n* = 30). The MAP and ROT were assessed before and after the 8-week intervention period.

**Results:**

The intervention group showed the significant reduction in MAP and ROT following the 8-week intervention period (*p* < 0.05).

**Conclusion:**

Daily consumption of seven Ajwa dates has a remarkable potential to decrease the MAP and ROT in pregnant women at risk of developing preeclampsia, and thus, it can contribute to prevent the development of preeclampsia.

## 1. Introduction

Based on the World Health Organization (WHO) reports in 2018, 830 deaths of women were reported every single day, due to pregnancy and childbirth complication. The maternal mortality arised from the problems in pregnancy and childbirth. The most fatal complications developed in pregnant women are severe bleeding, infection, hypertension in pregnancy (preeclampsia and eclampsia), and unsafe abortion [1]. In Indonesia, hypertension becomes one of the leading causes of maternal death. Hypertension in pregnancy may include chronic hypertension, preeclampsia, and eclampsia. Of these, preeclampsia is the major problem and the main leading cause of fetal and maternal mortality worldwide [2].

Preeclampsia is a syndrome characterized by the elevation of systolic blood pressure (≥140 mmHg), or diastolic blood pressure (≥90 mmHg), persistent after 20 weeks' gestation in pregnant women previously having normal blood pressure. The condition can be accompanied with or without the new onset of proteinuria. It is also associated with other signs and symptoms, including blurry vision, stomachache, headache, and edema. Preeclampsia, a syndrome in pregnant women, can result in complication for maternal and fetal lives. The maternal complications may include cardiovascular disease, cerebrovascular disease, liver and renal failure, placental abruption, and disseminated intravascular coagulation. However, the fetal complication can occur in terms of mortality and disability, resulting from fetal growth restriction, preterm birth, low birth weight, severe birth asphyxia, stillbirth, and intrapartum death. High adverse effect of preeclampsia and the lack of understanding in its pathophysiology encourage the need for early prediction of preeclampsia in pregnant women; therefore, various prevention methods to delay the progressivity of preeclampsia can be ensued. The study revealed that the combination of body mass index, MAP, and ROT can be used as prediction factors for the development of preeclampsia in pregnant women [3–5]. The date fruit, *Phoenix dactylifera L.* is one of the fruits which has abundant benefits for health, including as antioxidant, anti-hyperlipidemic, and hepatoprotective agent, and prevents cardiovascular diseases. The metabolic analyses were conducted for 12 kinds of dates which originated from Saudi Arabia to determine the nutritional composition in each kind of dates. It revealed that the highest level of phenolin was observed in Ajwa dates, while the highest flavonoid was found in Ajwa dates and then in Saffawi dates. However, the potential of Ajwa dates was not fully elucidated in pregnant women; thus in the present study, we aimed to evaluate the effect of daily consumption of seven Ajwa dates on prevention of preeclampsia, through MAP and ROT changes.

## 2. Materials and Methods

### 2.1. Study Population and Design

The randomized controlled study was conducted in Sitti Khadijah 1 Muhammadiyah Hospital, Makassar, South Sulawesi, Indonesia, from 28 January through 30 March 2019. The research protocols in the present study were approved by the Ethical Committee of Medical Faculty, Hasanuddin University (64/UN4.6.4.5.31/PP36/2019). The written informed consent was obtained from all participants. The inclusion criteria were as follows: (1) the pregnant women having one of the preeclampsia risk factors, including obesity, primipara, history of hypertension, preeclampsia history in previous pregnancy, and family history of preeclampsia; (2) pregnant women having predicted preeclampsia in one or both biophysical predictor assessment (MAP and ROT); (3) gestational age at ≥20 weeks; and (4) agreed to be included as study participants and then the informed consent was obtained. The exclusion criteria included (1) having fasting blood glucose and 2-hour postprandial blood glucose beyond normal range, (2) having previous history of chronic infection diseases (i.e., tuberculosis, malaria, and thalassemia), and (3) having degenerative diseases (i.e., cardiovascular diseases, cancer chronic renal failure, and diabetes mellitus). The screening was performed to include the participants meeting the established criteria in this study. The included participants in the present study were then randomly assigned into the control group (*n* = 10) and the intervention group receiving the daily intake of Ajwa dates (*n* = 30).

### 2.2. Experimental Procedure

At the beginning, the participants in the intervention group were given a counselling session. In the counselling session, the researchers provided information on risks of preeclampsia, the ways to manage the risk to prevent preeclampsia, and the benefits of daily consumptions of 7 pieces of Ajwa dates every single morning for general health and pregnancy. To reinforce the given information, the leaflet was provided to the intervention group. It consisted of information on preeclampsia and Ajwa dates. The participants in the intervention group were given the diary sheet to record their daily intake of Ajwa dates for eight weeks. Forty-nine Ajwa dates were provided per week to the intervention group. However, in the control group, the counselling session was conducted to inform the participants on the risks of preeclampsia and the ways to manage the risk to prevent preeclampsia. The control group was also encouraged to consume nutritious food, avoid fast food, and increase the consumption of fruits (except dates) and vegetables. The leaflet was provided to reinforce information on preeclampsia and nutrition need for pregnant women.

### 2.3. Outcome Measures

The measurements of MAP and ROT were performed prior to undergoing the intervention and at the completion of the 8-week intervention period in all participants in both groups. The blood pressure was assessed using a digital sphygmomanometer. Before initiating the measurement, the participants were asked to sit and relax. The systolic and diastolic pressures were recorded, and the MAP was obtained using the following formula:

(1) $$\begin{array}{c} \text{MAP}=\frac{\left(2\times \text{diastolic pressure}\right)+\text{systolic pressure}}{3}. \end{array}$$

ROT was performed by measuring the blood pressure in the side lying position and supine position. The ROT value was obtained based on the ROT formula.

### 2.4. Statistical Analysis

All data were presented as means ± SE. The data obtained in this study were analysed using the Wilcoxon-signed rank test. All statistical analyses were carried out using SPSS 22.0 software. A *p* value less than 0.05 was regarded as statistically significant.

## 3. Results

In the present study, we aimed to evaluate the potential effect of Ajwa dates on prevention of preeclampsia threats in pregnant women at risk of preeclampsia. The baseline characteristics of the participants are presented in Table 1. As shown in Table 2, the MAP and ROT were significantly improved in the intervention group, while the ROT changes were not statistically significant in the control group. However, the MAP in control group was significantly increased following the 8-week intervention period.

**Figure 1:**
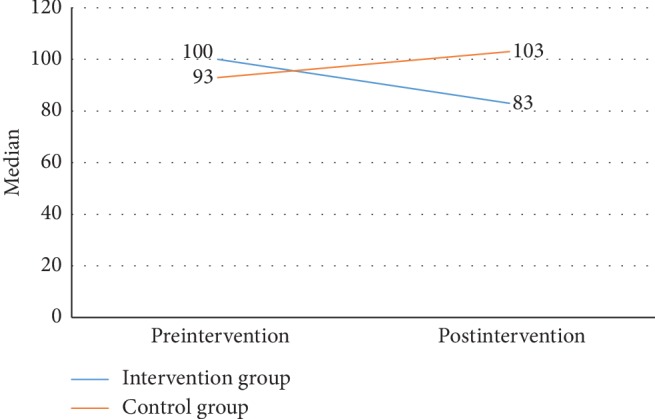
The median value of MAP changes in two groups.

**Figure 2:**
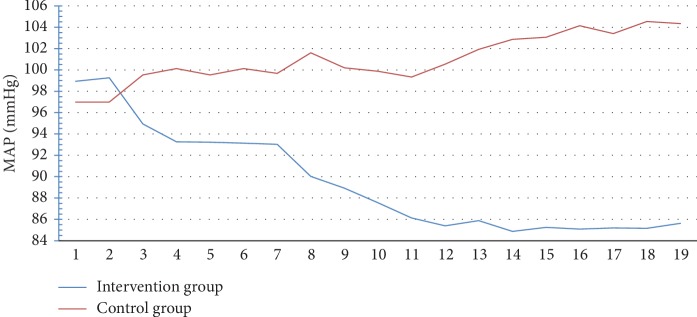
MAP changes every 3 days for 8 weeks in both groups.

**Figure 3:**
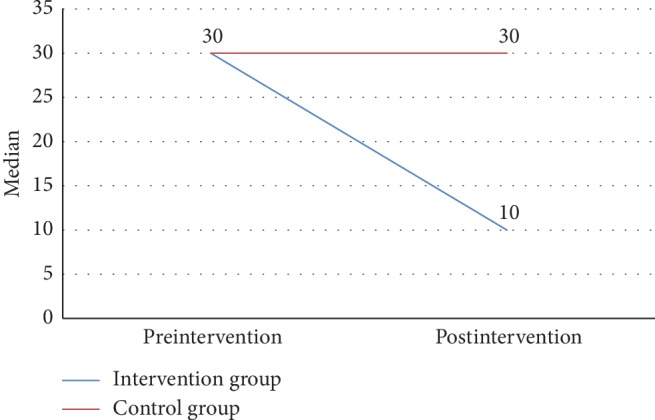
The median value of ROT changes in two groups.

As shown in Figure 1, the median value of MAP was decreased following the 8-week intervention period in the intervention group, while the inverse finding was shown in the control group. Progressivity of preeclampsia based on MAP did not occur in the intervention group, but not in the control group. To monitor the general health of participants, the blood pressure was assessed periodically once per 3 days. The changes in MAP in both groups every 3 days during 8 weeks are shown in Figure 2. In addition, the changes in ROT following the 8-week intervention period in terms of median values are shown in Figure 3. The ROT decreased in the intervention group, while an increase in ROT was seen in the control group. It indicates that the progressivity of preeclampsia was prevented in the intervention group, but not in the control group.

## 4. Discussion

The current study revealed that daily consumption of 7 pieces of Ajwa dates in pregnant women with preeclampsia risk had a potential to prevent the occurrence of preeclampsia through improvement in MAP and ROT. Regarding the amount of Ajwa dates, the study on daily consumption of seven dates was also conducted by Al Kuran et al. to evaluate its effect on labour parameters and delivery outcomes [6]. The same number of dates was also used in several studies to determine its effect on the labour process in pregnant women [7–9].

It has been reported that positive MAP at the first trimester in pregnant women can become the predictor of gestational hypertension and preeclampsia with detection rates being 76%. The probability of pregnant women with positive MAP to develop hypertension in 27 weeks of gestational age is 3,381 times compared to those with negative MAP [10, 11].

The main finding of the present study is that, on day 21, the significant decrease in MAP was shown in pregnant women regularly consuming Ajwa dates, while the inverse findings were shown in the control group. At the completion of the 8-week intervention group, the MAP decreased by 13%, while 8.1% increase in MAP was observed in the control group. The similar findings were shown on ROT values, in which 66.4% of reduction was observed in the control group, while 5.6% increase was shown in the control group. The mechanism underlying the potential of Ajwa dates in improving MAP and ROT might be due to the presence of potassium and magnesium in the Ajwa dates. Potassium and magnesium are the important minerals which play a role in controlling blood pressure, normal heart rhythm, and muscle contraction. The contained potassium also has function as a main regulator of blood vessel to maintain the elasticity of arterial walls which prevent the deterioration of vascular under high blood pressure. The other study revealed the reduction of systolic blood pressure by 14.23 mmHg due to the potassium contained in dates (Deglet Nour). In addition, high level of potassium and low level of sodium in Ajwa dates are seemingly suitable for individuals with hypertension. Furthermore, the magnesium in Ajwa dates also functions to activate the Na+/K+ pump, which results in lowering of diastolic blood pressure. Hence, dates have become the traditional therapeutic approach for hypertension in Maroko [12–14].

On the contrary, vasodilator activity of flavonoid contained in Ajwa dates also contributes to reducing the blood pressure. As we know, endothelial dysfunction has a complex relationship with the hypertension, preceding the development of pathologic condition in the cardiovascular system. Therefore, improving the endothelial function may contribute to decrease the blood pressure. High consumption of flavonoid-rich foods can prevent the cardiovascular disease which has been proven in the pharmacological and epidemiological study [15–19]. The similar findings on the antihypertensive activity of Ajwa dates were also reported by Qureshi and Khan [20].

## 5. Conclusions

Based on the findings in the present study, we concluded that daily consumption of seven Ajwa dates has a remarkable potential to decrease the MAP and ROT in pregnant women at risk of developing preeclampsia, and thus it can contribute to prevent the development of preeclampsia.

## Figures and Tables

**Table 1:** Baseline characteristics in both groups.

Variables	Control group (*n* = 10)	Intervention group (*n* = 30)
Age (year)		
Low risk	7 (70%)	25 (83.3%)
High risk	3 (30%)	5 (16.7%)

Educational level		
Primary school	0 (0%)	6 (20%)
Junior high school	5 (50%)	5 (16.7%)
Senior high school	3 (30%)	12 (40%)
Diploma	0 (0%)	4 (13.3%)
Bachelor	2 (20%)	3 (10%)
Gestational age (mean ± SD)	22.02 ± 2.04	22.9 ± 2.95

Gravida		
1^st^	6 (54.5%)	14 (46.7%)
2^nd^	2 (18.2%)	7 (23.3%)
3^rd^	2 (18.2%)	8 (26.7%)
4^th^	1 (9.1%)	1 (3.3%)

Employment status		
Employed	7 (60%)	21 (70%)
Unemployed	3 (30%)	9 (30%)

**Table 2:** Outcome measurement in two groups.

Outcome	Control group (*n* = 10)			Intervention group (*n* = 30)		
	Before	After	*p* value	Before	After	*p* value
MAP	96.70 ± 4.85	104.3 ± 3.60	0.003^*∗*^	98.9 ± 4.41	85.03 ± 4.38	<0.001^*∗*^
ROT	30.40 ± 2.27	32 ± 4.22	0.223	30.13 ± 2.16	10.07 ± 8.09	<0.001^*∗*^

MAP, mean arterial pressure; ROT, roll-over test. ^*∗*^*p* < 0.05, statistically significant.

## Data Availability

The raw (excel) data used to support the findings of this study are available from the corresponding author upon request.
